# The Action of the Voluntary Muscles

**Published:** 1862-05

**Authors:** Louis Mackall


### SELECTED.

### THE ACTION OF THE VOLUNTARY MUSCLES.

By LOUIS MACKALL, M. D.

[Extract from an unpublished work.]

69. The action of the Vountary muscles is regarded by the law of nature that we have already stated, (37.) By virtue of this law, the determination of the nerve-fluid* to a muscle is immediately followed by the active elongation of its fibres; the contraction of a muscle or of its fibres as immediately follows the withdrawal of the nerve fluid. In other words, the presence of the nerve-fluid, its cause of action, is, by virtue of this law, always attended by an active elongation of the fibres of a muscle; while the absence of a due portion of this fluid is as constantly attended by the contraction of its fibres.

By the aid of this law of nature, we have explained, I think, in a plain, intelligible, and rational manner, the phenomena presented in the action of the involuntary muscles, that are placed about the walls of the tubes or hollow organs; by the same aid, the phenomena presented in the action of the voluntary muscles may be as rationally and intelligibly explained.

70. The minds of Physiologists of the present day are so fully impressed with the erroneous belief, so long inculcated in the books, that the active state of a muscle is that of contraction, and the elongation of its fibres, (a state of which they possess a very imperfect and false notion,) which they are pleased to call relaxation, is a passive state, that the converse of this proposition, as presented above, will be at first regarded by them with prejudice and aversion. The true law of nature in relation to this subject, however, when its application is attended to, in the extensive application of it we propose to present, must in time be acknowledged; if truth, as is said, has a tendency to prevail over error in the human mind.

71. When we use the term muscle, it should be understood to embrace both the tendinous and fleshy portions of the organ; the same fibres, we conceive, extend through the whole, and are influenced by the cause of action, the nerve-fluid, as well in the tendon as in the fleshy part. The latter portion differs

* Why is this term still allowed to disfigure scientific medical writing ?—Ed. Jr.

from the former probably only in the circumstance of being more largly supplied with nerves and blood-vessels, by means of which, when the muscle is in a state of action, the special circulation of the nerve fluid (43) is established.

72. For the better illustration of their action, it will be found convenient to seperate the voluntary muscles into the four following classes, viz: 1st. Such as are attached by one extremity only, having the rest of the muscle free to be extended or retracted at will; 2d. Such as are attached at both extremities, but are capable only of the same motion as the former class, that of direct extension of retraction; 3d. Such as are arranged circularly about the openings of the organs, and serve as sphincters; and 4th. Such as are attached to the bones at both extremities, and act by making use of the bones as levers.

THE ACTION OF THE FIRST CLASS OF VOLUNTARY MUSCLES,

*That are attached by one extremity, and have the rest of the muscle free to be extended or extracted.*

73. Of the action of this class we will advert to that of the muscles of the tentacles in the lower orders of animals; and among the higher orders, to that of the muscles of the tongue, of the ciliary processes, and of the male Organ of generation.

Perhaps there is no other instance in nature, in which is presented so fairly and clearly, the true condition of a muscle when in a state of action, as in that of the action of the muscles of the organ last mentioned. Physiologists, by a resort to Sophistry, of which we shall speak again, have ignored the action of these muscles, which is the same with that of all other muscles; for the state of erection produced by the action of these muscles is common to all the muscles of the living body when in action. Every muscle when in action is in a state of vital erection; which term should be understood to express simply the active elongation of the fibres, with a due supply of blood and of nerve-fluid to maintain this state. In the Human subject, the true character of the organ of which we are speaking is masked, as it were, by the great development of the blood-vessels and nerves with which it is supplied; but this is not the case in some other species of animals, as in the Horse, the Ox, the Hog, and the Sheep. In these the main body of the organ is composed of longitudinal muscular fibres that may be actively elongated to a length of from twelve to twenty inches. The view of muscular action heretofore presented may be repeated in connection with the instance before us.

74. The suggestive impressions, appointed to precede the active state of this organ, having been duly received, the anima determines the nerve-fluid, in an extra supply, to its fibres through the sensory and motory nerves of this organ. In consequence, the proper longitudinal and other fibres of the organ become actively elongated, together with the fibres about the walls of the vessels and sinuses, in which there is an increased flow and accumulation of blood. With this accumulation of blood there is established the special circulation of the nerve-fluid to this point; the supply of this fluid is thus further enlarged, and the action of all the fibres is exalted to its extreme limit. This latter state of the organ is expressed by the term "Venereal orgasm.'

75. In this representation of the action of muscles, there are three points to which we desire to direct attention: the first is, the propriety of regarding the organ in question as a muscle attached at one extremity by two heads to the ossa pubis and ischia; the second is, *the active elongation* of the proper fibres of this muscle; and the third is, the *stiffening or rigidity* of the same fibres when in action. The two latter points are particularly worthy of attention, as they occur in every instance of muscular action, and what is very strange, have been entirely overlooked by physiologists.

76. The Ciliary processes are the small muscles or bundles of muscular fibres that are attached by one extremity to the margin or verge of the opening into the eye, called the pupil; and are so arranged that by their extension the pupil is diminished or the opening narrowed, and by their retraction the pupil is enlarged or the opening widened. Light is appointed to be the appropriate suggestive impression to precede the action of these muscles. When the eye, in its normal condition in the living body, is withdrawn from the light, the mind, not having received this suggestive impression, does not call into action these muscles, and they are consequently retracted, and the pupil is enlarged; but when the eye is brought into the light and the impression is made, the mind determines the nerve-fluid to these muscles, and they become extended in proportion to the supply of this fluid; by their combined action the pupil is diminished or narrowed, so as to exclude such quantity of light as might be injurious to the optic nerve.

Here again we have a Vital erection, in the essential conditions—the active elongation, and apparently the stiffening of the fibres; and it is worthy of remark here, that in inflammation of these and the adjoining parts, as in iritis, there is established the special circulation of the nerve-fluid to these fibres, and they become persistently elongated, and the pupil continues to be narrowed even when the eye is withdrawn from the light.

77. The longitudinal muscular fibres of the Tongue (with a view to the elucidation of the action of this organ) may all be regarded as one muscle, that is attached at one extremity, with the other parts free to be extended or retracted at will. The active elongation or extension of these fibres when innervated, or when in action is manifest in our own persons, if we confine our attention to the fibres in question; but this active elongation of the tongue in some of the lower orders of animals is very remarkable. Every one is familiar with the protrusion and elongation of the tongue of some of the domestic animals, as the Dog, the Cow, &c.

This action of the tongue of the Serpent is deserving of particular notice. When the animal is aroused or excited, it erects its head and trusts out of its mouth its forked tongue, to the extent of several inches. The suddenness and celerity of the motion of the organ forcibly reminds us of the appearance of a flash of lightning.

It is impossible, I believe, to suggest any rational explanation of this phenomenon, other than that of the innervation and consequent elongation of the fibres of the muscles that belong to this organ. The suddenness and celerity of the motion precludes all other agency but that of the nerve-fluid, passing along the nerves; which alone resembles in its motion that of the Electric fluid.

The action of the tongue of the Frog or toad, when it seizes its prey, is similar to that of the serpent.

78. A notable action of this kind is presented in that of the tongue of the Chameleon. To facilitate its description, this organ may be divided into four parts; first, the anterior bulbous portion, formed by the interweaving of cellular and muscular tissues—with the appearance of the end of the trunk of the elephant, it is furnished like this with a fleshy forceps at the extremity, with wich the animal seizes its prey; second, the middle or interior portion, *composed entirely of cellular tissue formed into a number of bands or hoops, having the intervals between them supplied with very loose and extensile meshes of this tissue;* third, the lingual bone, being a process from the centre of the os hyoides, arranged in the direction of the tongue, on which, when the tongue is retracted, the cellular bands and meshes of the second portion, and a part of the bulbous portion, which is hollowed out for this purpose, are stretched or drawn, like the finger of a glove drawn over the finger; and forth, two well-developed muscles, having their fibres arranged longitudinally, and largely supplied with nerves and blood-vessels. These muscles arise from the cornua of the Os hyoides, one from each, and being arranged one on each side of the tongue, or of the central cellular portion, are inserted in the bulbous extremity.

When in a state of repose, or when retracted within the mouth, the organ is from an inch to an inch and a half long; but when about to seize its prey, commonly an insect, the chameleon creeps to within seven or eight inches of the object, and then fixing its body, and taking aim, as it were, suddenly, and with the celerity of lightning, thrusts forward its tongue, and seizes the insect with the fleshy forceps at its extremity. The food thus taken is then drawn into the mouth by the retraction of the organ.

79. If what we have suggested be admitted to be the true law of muscular action—if innervation, or the determination of the nerve-fluid to a muscle, is attended with the active elongation of its fibres, there is no difficulty whatever in comprehending or in offering a rational explanation of the phenomenon before us. The animal, it will be understood, determines its nerve-fluid to the lingual muscles, and the tongue is extended to the distance mentioned, simply by the active elongation of these muscles. The food is also drawn into the mouth by the retraction of the same muscles, caused by the withdrawal from them of this fluid.

But what explanation of this phenomenon can be given without the aid of this law? We give the only one we have met with worthy of any notice, that of Mr. John Hunter, in his own words, taken from the "Illustrated Catalogue of the Hunterian Museum."

"This length of tongue, its extension and contraction, are very singular, and if we well understood, very probably very curious.

"The cause and mode of the contraction of its length are not uncommon. The elongation of the tongue in this animal is perhaps like nothing that we are acquainted with in an animal body.

"The apparatus for this purpose is a small rounded body which passes from the apex of the os linguæ (glosso-hyal) to the bulbous part, and then through the centre of the bulb. The part between bone and bulb consists of two different substances, one a whitish substance, which is the firmest, and appears to be capable of keeping its form; the other is softer and more transparent. That part which passes through the bulb consists only of one substance, and appears to be a sheath for the reception of the os linguæ." The reader will please recollect that the apparatus, here described by Mr. Hunter, is nothing more than the bands and meshes of *cellular tissue* of which we have spoken above. But let him proceed:

"The first of these (*i. e.* the whitish, firmer substance) appears to be composed of rings or something similar placed obliquely in contrary directions, so as to appear to be two spirals crossing one another. Whether the other or softer substance" (the cellular meshes, L. M.) "has any direction of fibres I could not observe, but I suspect it is muscular. If I am right in my conjecture of this structure, and of its disposition, it will be no difficult thing to show how it may be elongated; for if these rings are placed transverse, they may be brought so near to one another as to shorten the whole very considerably; and if they allow of being placed almost longitudinally, they must of course lengthen it very considerably, and this position can easily be produced by muscles, which I take the pulpy substance to be.

"The contraction of the tongue is owing to a degree of elasticity; but this appears to be only in the cellular membrane, acting as an assistant to the muscular. The muscular contraction is owing to two muscles, one on each side of the tongue; each arises from the os hyoides on the inside of the os linguale, and passes along the side of the tongue to its bulbous part; but before it gets to the bulbous part it spreads itself all round.

"In the centre of each of these two muscles passes a considerable nerve to the bulbous part, and also two arteries. When the two muscles act, they draw the tongue back upon the os linguale, which, as it were, passes through the middle elongator, then through the centre of the bulb, till the whole tongue is retracted. Although the middle body is drawn upon the os linguæ, yet it does not appear to be a hollow like a pipe; it rather appears to be filled with a very ductile cellular membrane, as in every part of the elongating division of the tongue, in order to allow of the great difference in the situation of parts with respect to one another." (Hunterian manuscript.)

80. The Ant-eater (Myrmeco-phaga) furnishes a remarkable instance of the action of the muscles of this class. The tongue of this animal is composed of two distinct muscles having different origins, and a different disposition of fibres. To simplify the subject, we may say one of these muscle arises from the sternum, and passing along through the muscles on the anterior surface of the neck, terminates at the tip of the tongue—its fibres being all arranged longitudinally; the other muscle arises from the os hyoides, passes around the former longitudinal muscle in close spirals, forming a sort of case or sheath for it, and terminates with it, at the end of the tongue.

When quiescent, or in a state of inaction, the tongue of the Ant-eater is probably about six or eight inches long; but when thrust into the ant-hills to secure its prey, Naturalists tell us, it is elongated or protruded to the extent of *seventeen* or *eighteen* inches.*

The object attained by this curious arrangement of the muscles, in accordance with our view of muscular action, is evidently, the free and unimpeded extension of the longitudinal muscle to its full capacity, by preserving it fibres, nerves, and blood-vessels from external pressure, while threading the narrow passages of the ant-hill through which it is forced. This object is fully accomplished by the action of the spiral muscle, which tends to dilate these passages, or at least, to enlarge and keep open the space enclosed by it, in which the longitudinal muscle acts.

In the absence of this view, Physiologists have been forced to adopt the very absurd supposition, that the tongue of the Ant-eater is protrude by the *contraction* of its *spiral muscle!*

81. The Woodpecker, as is well known, feeds on the grubs that burrow into the limbs and bodies of trees. The burrows are first exposed by pecking through the bark with its strong, sharp bill, and then the long tongue, like a flexible probe, is thrust in, as the Ant-eater's into the ant-hill, and the grub being harpooned, as it were, is dragged forth.

To adapt the organ to the purposes mentioned, the anterior portion of the tongue—I refer more particularly to that of the species called the Flicker, (Picus aureatus)—is composed of a horney, spear-shaped, barbed point; from this point there arise two muscles, tendinous at each extremity, but with fleshy bellies or middles—their fibres longdituinal; these muscles pass around the base of the cranium, one on each side, and then rest with free extremities in a groove or closed passage formed between the skin and cranium, and extending over the

* Curvier's Animal Kingdom, edited by Griffith, vol. iii, p. 300.

middle of the head from behind forward until it reaches the base of the upper Mandible. Besides these, two other muscles arise, one from the ramus of the lower mandible on each side, and pass around the longitudinal muscles, forming a sheath for them through their whole length. The arrangement of these muscles are strikingly similar to that of the muscles of the tongue of the ant-eater, and doubtless their functions are the same. When the longitudinal muscles are innervated and elongated, the upper bill, which is then applied to the tree, becomes the basis or part of resistance, from which the tongue is protruded into the burrows; the other or spiral muscles acting, as suggested, when speaking of that of the ant-eater, to preserve the fredom of the motion of the former, by protecting from external pressure the blood-vessels and nerves with which they are largely supplied.

The notion suggested by some, that the tongue is jerked forward by the contraction of the last-mentioned muscles, is absurd; as the means suggested are clearly inadequate to the end proposed. The contraction of any portion of these muscles could not effect a motion of the tongue to the extent of one inch, but, if contracted, the portions anterior to their origins would draw the tongue back, and counteract the effect of the contraction of the portions of these muscles that are posterior to their origins, so that no effect of the kind suggested, could result from the contraction of these muscles. The tongue is thrust into the burrows, probably to a distance of from five to eight inches, judging from the extensibility of the organ in the living or recently killed bird.

The longitudinal muscles of the tongue are commonly regarded as the cornua of the os hyoides, but I think it more rational to regard them as muscles (as their appearance clearly indicates) belong to this class, attached at one extremity with the rest of the organ free, to be extended or retracted at will.

82. The "Aij-Aij" is an animal whose habitat is confined to Madagascar. It presents several points in its natural history closely resembling those of the woodpecker. Like this, it feeds on a grub that burrows in the bodies of trees in that island; and it exposes these burrows by tearing off the bark with its teeth. The animal then introduces a long flexible probe, and pulls out the grub. This probe, however, is not the tongue; but one of the fingers of one of the fore-paws, which is curiously adapted in its structure to this purpose. The anatomy of this organ is not generally known, but I presume, from the office it is said to perform, that it is similar to that of the tongues of the Ant-eater and of the Woodpecker that we have just described. In its retracted state it may present the appearance of a "shrunken" or "atrophied" member, as is represented; but its muscles are, I doubt not, well developed, with large blood-vessels and nerves, and capable of an extensive action of elongation.

83. The Tentacles of some of the lower orders of animals are so similar in structure, in their action, and in the purposes they subserve, to the tongues of the higher orders, that we are induced to regard them as their analogues. These organs are composed of muscles with longitudinal fibres, that are attached by one extremity around the outer margin of the mouth, and are extended or actively elongated for the purpose of collecting and of bringing into the mouth, the food or prey that may be floating in the element these animals inhabit. When the animals are reposing, or not feeding, these organs are retracted; but when engaged in taking their food, the tentacles are innervated, and actively elongated or extended. In some instances, as in the Physalus, the Enychoteuthis, &c., this extension is carried to a distance of six feet or more.* For a more minute account of the structure and office of these organs, I refer to the works on comparative Anatomy and Physiology. Our theory of muscular action offers to the intelligent student a number of suggestions that will be found of great use to him in his efforts to understand the more complicated structure and action of some of these organs.

[The remainder of this valuable extract, kindly submitted for our disposal by the Author, will be published in the next number of this journal.—Ed.

### PROSECUTION FOR INFANTICIDE.

Dr. H. Culbertson, of Zanesville, Ohio, in the "*Lancer & Observer*," after giving a condensed statement of the evidence in a recent trial for infanticide, offers the following valuable remarks.

We regret that our limits will not permit the full details of the case, with Dr. C.'s opinion, and its reasons, on the sanity of the respondent. The prisoner having been acquitted under

* General Structure of the Animal Kingdom, by T. Rymer Jones.

